# Current decline in schistosome and soil-transmitted helminth infections among school children at Loum, Littoral region, Cameroon

**DOI:** 10.11604/pamj.2019.33.94.18265

**Published:** 2019-06-10

**Authors:** Shanti Merveille Mekam Nkengni, Aurelia Thérèse Condomat Zoumabo, Naomi Paloma Sangue Soppa, Adèle Besch Ngwem Sizono, Philippe Vignoles, Louis-Albert Tchuem Tchuenté, Félicité Flore Djuikwo Teukeng

**Affiliations:** 1 Faculty of Health Sciences, Campus of Banekane, Université des Montagnes, P.O. Box 208, Bangangté, Cameroon; 2 INSERM U 1094, Institute of Neuroepidemiology and Tropical Neurology, 2, rue du Docteur Raymond Marcland, 87025 Limoges, France; 3 Centre for Schistosomiasis and Parasitology, Texaco Omnisport and Laboratory of Parasitology and Ecology, Faculty of Sciences, University of Yaoundé I, Yaoundé, Cameroon

**Keywords:** Cameroon, Loum, schistosoma haematobium, s. mansoni, schistosome, soil-transmitted helminth

## Abstract

**Introduction:**

Soil-transmitted helminth infections (STHs) and schistosomiasis have serious consequences for the health, education and nutrition of children in developing countries. As Loum is known as a highly endemic commune for these infections, several deworming campaigns have been carried out in the past. The purpose of this study was to determine any changes that have occurred since then in the characteristics of these infections among schoolchildren in this site.

**Methods:**

A cross-sectional study was conducted in October 2016 on 289 schoolchildren. Stool and urine samples were collected and examined to determine the prevalence and intensity of helminth infections.

**Results:**

The highest prevalence was noted for *Schistosoma haematobium* (34.2%), followed by *Ascaris lumbricoides* (8.6%), *S. mansoni* (4.9%) and *Trichuris trichiura* (4.9%) in decreasing order. A prevalence of less than 2% was noted for each of the other two helminths. The highest mean intensity was found for *S. haematobium* (39.6 eggs/10 ml of urine), followed by *A. lumbricoides* (24.2 eggs per gram of faeces: epg), *Strongyloides stercoralis* (16.6 epg) and *Schistosoma mansoni* (12.3 epg). The prevalence of *T. trichiura* was significantly higher in boys and that of *S. haematobium* in children aged 10 years or older, while the differences between other values of prevalence or between egg burdens were not significant.

**Conclusion:**

Compared with values reported in 2003, the prevalence and intensity of *schistosomiases* and STH infections in Loum has sharply decreased in 2016. Confirmation of this decrease in the years to come allowed to space deworming campaigns among schoolchildren.

## Introduction

Schistosomiasis and soil-transmitted helminthiases (STHs) are among the most common infections in humans living in tropical and subtropical countries and where sanitation is rare. They remain public health problems in the poorest developing world and are mostly found in rural areas of low- and middle-income areas [1]. Lack of hygiene and certain play habits of school-aged children such as swimming or fishing in contaminated waters make them especially vulnerable to schistosome infections [2]. Among STH infections, *Ascaris lumbricoides, Trichuris trichiura* and hookworms are of particular importance to public health as they have a negative impact on the health, education, nutrition and economic development of populations. These infections affect the poorest and most deprived communities, and are transmitted by eggs and larvae in human faeces, which contaminate the soil in areas of poor sanitation [2, 3]. According to Pullan *et al.* [4], 819.0, 464.6 and 438.9 million people are infected with *A. lumbricoides, T. trichiura* and hookworms, respectively. Human mortality caused by these STH infections is rare. In contrast, the morbidity and the harmful effects of these infections on socio-economic development are enormous [5, 6]. Control of schistosomiasis and STH infections is focused on disease reduction through large-scale mass drug administration (MDA) programs implemented periodically and on prevention of people through education for the population health, drinking water, adequate sanitation and personal hygiene [7, 8].

In Cameroon, schistosomiasis and STH infections are important parasitic diseases. More than 5 million people are at risk of schistosomiasis and 2 million people are currently infected with *Schistosoma haematobium* or *S. mansoni.* Two species of STH, *A. lumbricoides* and *T. trichiura*, are widely distributed throughout the country and it is estimated that more than 10 million people are infected with gastrointestinal worms [9-11]. The highest levels of schistosomiasis transmission were observed in northern Cameroon, whereas STH infections were more prevalent in the southern part [12, 13]. School-aged children were the most infected and polyparasitism very common, with a high proportion of children carrying at least two parasite species [9]. Since 2003, some campaigns for helminth detection and deworming have taken place throughout the country. School-aged children were treated with mebendazole throughout the country, while Praziquantel was only distributed in areas of high endemicity for schistosomiasis. According to Tchuem-Tchuenté *et al.* [11], transmission of schistosomiasis increased in several health districts, while there was a significant decline in STH infections. These authors recommended continuation of annual or biannual mass drug administration (MDA) campaigns for STH infections and extension of Praziquantel in communities identified as moderate to high risk for schistosomiasis. In view of these findings, it was interesting to determine the current state of these parasitoses in a highly endemic area via the following two questions: were the prevalence values of schistosomiasis and STH infections decreasing? What was the parasitic load of these helminths in schoolchildren? To answer these two questions, stool and urine samples were collected in October 2016 from 289 schoolchildren in the commune of Loum (West Cameroon). These samples were examined to detect helminth species and count their eggs. In a second step, the results obtained will be compared with those reported by Tchuem-Tchuenté *et al.* [14] for the children of five schools of Loum.

## Methods

### Study area

The town of Loum (4°43' N, 9°43' E) is located in the department of Moungo about 70 km from Douala and extends over a plateau with an average altitude of 180 m to 235 m. The city is surrounded by high mountains, hills and plateaus. It is crossed by the river Mbette which takes its source on Mount Koupé (2070 m) located to the north of the city. The soils are of volcanic origin and black texture over a large part of the communal territory. The climate is equatorial with a wet season of about 9 months (March to November) and a short dry season (December to February). Precipitation is abundant (a mean of 2699 mm per year), while the mean monthly temperature fluctuates from 19°C to 25°C. This city is known as a high endemic zone for schistosomiasis and several authors have already taken urine or stool samples from schoolchildren in this city over the past forty years to study schistosome infections [14-16]. According to Tchuem-Tchuenté *et al.* [14], the main sites of schistosomiasis transmission have been identified in areas adjacent to the Mbette river and its tributaries.

### Study design

In Loum, two public primary schools (CEBEC A, SANDA 2) were chosen for three reasons: a) schoolchildren belong to the age group of the most affected population; b) individuals in this age group are less hesitant to undergo mass examinations; c) these results could be compared with those of previous studies because they were performed on the same school population. Two other criteria led us to select these institutions: 1) the distance between each school and the Mbette River to check whether the proximity of the school or its distance from the river affected the prevalence of parasitic infections in children, and 2) the strong awareness of teachers and parents/guardians to the problems caused by polyparasitism in children. The sample size was calculated using the following formula [17]:

$$x=\frac{Z^{2}*\text{p}(1-\text{p})}{{\text{e}}^{2}}$$

With Z, constant value (1.96); p, prevalence of urinary schistosomiasis at Loum (40.27% [14]); e, effect size for an acceptable accuracy of 0.06. According to this formula, at least 255 schoolchildren were necessary for this study. A sample of 289 children, including 151 boys and 138 girls aged 4 to 15 years, was thus used. Urine samples were collected from these 289 children. In contrast, stool samples were less numerous (245 only) because 44 children did not want to give their stools. The parents or guardians of these 289 children have given their written consent for this study and no anthelmintic treatment was taken by the children during the collection of their urine and/or stool. Children from villages around Loum and those without the written consent of their parents or guardians were excluded from this study.

### Sample collection and examination

Stool and urine samples were collected each morning from each participant and were placed in 60 mL screw-cap vials with indication of gender, age of the child and school. These samples were stored in a portable cooler and transported to the laboratory at the Université des Montagnes for examination on the same day. The two techniques used were those that Tchuem-Tchuenté *et al.* [14] used in their study. Each urine sample was shaken vigorously to ensure adequate dispersion of eggs and 10 mL were filtered through a Whatmann^®^ Nuclepore membrane. The filter was then examined to detect the presence of *S. haematobium* eggs. Stool samples were examined by the thick smear technique using a 41.7 mg Kato-Katz template and each slide was read immediately after its preparation. Parasite eggs were identified according to the keys by Thienpont *et al.* [18] and Kaufmann [19]. The results were expressed as the numbers of helminth eggs per 10 mL of urine (*S. haematobium*) or per gram of faecal matter (epg) for each species of helminth found in the stools.

### Ethical considerations

The present study was approved by the National Ethics Committee of Cameroon (No. 2016/078/UdM/PR/CAB/CIE), the Ministry of Public Health and the Ministry of Basic Education. Parasitological surveys were conducted in both schools with the approval of local administrative authorities, school inspectors, directors and teachers. Information about the National Programme for the Control of Neglected Tropical Diseases and the objectives of this study were explained to schoolchildren and their parents or guardians.

### Parameters studied

The prevalence of each helminth infection was calculated using the ratio between the number of infected schoolchildren and the total number of children involved in this study. Fisher’s exact test was used to study the relationships between the prevalence of infections and the gender of children, their sex or their school. Four age groups were considered: <7 years, from 7 to 9 years, from 10 to 12 years and >12 years. All prevalence values are given with their 95% confidence intervals. The second parameter was the intensity of each helminth infection, measured by the number of eggs. Individual values noted for this last parameter were averaged and the standard deviations calculated taking into account the parasite species. The normality of these intensities was analyzed using Shapiro-Wilk test [20]. As the distribution of these values was not normal, the Scheirer-Ray-Hare test was used to establish levels of significance. All these analyses were performed using software R 3.3.0 [21].

## Results

### Prevalence of helminth infections

Five species of helminths: *A. lumbricoides, S. mansoni, Strongyloides stercoralis, Taenia saginata* and *T. trichiura*, were noted in stool samples of these children. In urine samples, *Schistosoma haematobium* was the single parasite found in the urine samples. Of the 289 schoolchildren, 125 were infected with one or more parasite species, so the overall prevalence of these infections was 43.2%. The highest prevalence (34.2%) was noted for *S. haematobium*. In the case of STH infections, the prevalence was 8.6% for *A. lumbricoides*, 4.9% for *S. mansoni*, 4.9% for *T. trichiura*, 1.6% for *Taenia saginata*) and 1.2% for *Strongyloides stercoralis* (Table 1). No hookworm eggs were found in these stool samples.

**Table 1:** Prevalence of STH infections and schistosomiasis in the schoolchildren of Loum in relation to their gender

Helminth species	Number of infected children/total number of children (Prevalence in %) [95% confidence interval]		
	Boys	Girls	Total
Stool samples			
*Ascaris lumbricoides*	10/132 (7.58) [3.69;13.49]	11/112 (9.83) [5.00;16.89]	21/245 (8.61) [5.40;12.86]
*Schistosoma mansoni*	8/132 (6.06) [2.65;11.59]	4/112 (3.57) [0.98;8.89]	12/245 (4.92) [2.56;8.43]
*Strongyloides stercoralis*	0/132 (0)	3/112 (2.68) [0.56;7.63]	3/245 (1.23) [0.25;3.55]
*Taenia saginata*	2/132 (1.5) [0.1;5.3]	2/112 (1.7) [0.2;6.3]	4/245 (1.6) [0.4;4.1]
*Trichuris trichiura*	10/132 (7.58) [3.69;13.49]	2/112 (1.79) [0.22;6.30]	12/245 (4.92) [2.56;8.43]
**Urine samples**			
*Schistosoma haematobium*	56/154 (36.36) [28.70;44.49]	42/132 (31.82) [23.90;40.49]	99/289 (34.27) [28.70;40.08]

The prevalence of *T. trichiura* was significantly higher (*p* = 0.041) in boys than in girls, while there was no significant difference between the prevalence of other parasites and the sex of children (Table 1). The distribution of infected children in relation to the four age groups is shown in Table 2. The prevalence of *S. haematobium* infection was significantly higher (*p* = 0.028) in children aged 10 years or older, whereas there was no significant difference in prevalence values for the five other species of parasites in relation to the age of children. No significant difference between the prevalence values of each helminth infection was noted when the school factor is taken into account (data not shown).

**Table 2:** Number of infected children and prevalence of infection in 289 schoolchildren (S. haematobium) or 245 (the other five species) in relation to their age

Helminth species	Number of infected children/total number of children (Prevalence in %) [95% confidence interval]			
	<7 years	7-9 years	10-12 years	>12 years
*Ascaris lumbricoides*	6/30 (20.0) [7.71;38.5]	8/91 (8.79) [3.80;16.59]	5/105 (4.76) [2.56;20.76]	2/19 (10.33) [1.30;33.14]
*Schistosoma haematobium*	7/32 (21.88) [9.27;39.97]	31/113 (27.43) [19.50;36.66]	51/123 (41.46) [32.60;50.69]	10/23 (47.62) [25.70;70.22]
*S. mansoni*	2/30 (6.67) [0.82;22.07]	8/91 (8.79) [3.80;16.59]	2/105 (1.90) [0.23;6.71]	0/19 (0)
*Strongyloides stercoralis*	0/30 (0)	0/91 (0)	2/105 (2.90) [0.23;6.71]	1/19 (5.25) [0.13:26.03]
*Taenia saginata*	0/30 (0)	3/91 (3.33) [0.69;9.33]	1/105 (0.95) [0.02;5.19]	0/19 (0)
*Trichuris trichiura*	2/30 (6.67) [0.82;22.07]	2/91 (2.20) [0.27;7.72]	6/105 (5.71) [2.12;12.02]	2/19 (10.53) [1.30;33.14]

### Polyparasitism

Two helminth species were noted in 14 schoolchildren. Four of them were infected with *A. lumbricoides* and *S. haematobium*, two with *A. lumbricoides* and *S. mansoni*, two with *A. lumbricoides* and *T. saginata*, and the two others with *A. lumbricoides* and *T. trichiura*. *Schistosoma haematobium* was noted in the other six children in association with *S. mansoni* (2 children), *T. trichiura* (2) and *T. saginata* (1). A triple infection with *A. lumbricoides*, *S. haematobium* and *T. trichiura* was noted in a single child.

### Intensity of snail infections

The highest number of eggs (Table 3) was found for *S. haematobium* (a mean of 39.6 eggs/10 ml of urine), followed by *A. lumbricoides* (24.2 epg), *S. stercoralis* (16.6 epg) and *S. mansoni* (12.3 epg) in decreasing order. The mean intensity in stool samples was 7.7 epg and 10.0 epg for *T. saginata* and *T. trichiura*, respectively. Table 3 and Table 4 show the numbers of helminth eggs in the urines and stools of children in relation to their gender and age, respectively. All the differences between these intensities of infections were not significant, whatever parasite species and the factor studied.

**Table 3:** Number of helminth eggs found in the infected children of Loum in relation to their gender

Helminth species	Number of helminth eggs: m ± S.D. (number of infected children)		
	Boys	Girls	Total
*Ascaris lumbricoides*	28.0 ± 46.6	22.6 ± 27.5	24.2 ± 38.0
*Schistosoma haematobium*	44.7 ± 47.7	32.7 ± 28.1	39.6 ± 40.6
*S. mansoni*	13.5 ± 5.4	10.0 ± 3.7	12.3 ± 5.0
*Strongyloides stercoralis*	0	16.6 ± 7.2	16.6 ± 7.2
*Taenia saginata*	5.5 ± 0.7	10.0 ± 2.8	7.7 ± 3.0
*Trichuris trichiura*	10.0 ± 3.5	10.5 ± 3.5	10.0 ± 3.1

**Table 4:** Number of helminth eggs found in the infected children of Loum in relation to their age

Helminth species	Number of helminth eggs: m ± S.D. (number of infected children)			
	<7 years	7-9 years	10-12 years	>12 years
*Ascaris lumbricoides*	24.1 ± 30.6	18.1 ± 20.6	38.6 ± 70.1	13 ± 1.4
*Schistosoma haematobium*	37.8 ± 29.9	41.1 ± 31.0	41.3 ± 48.8	27.9 ± 27.1
*S. mansoni*	18.0 ± 5.6	11.5 ± 4.9	10.0	0
*Strongyloides stercoralis*	0	0	18.5 ± 9.1	13.0
*Taenia saginata*	0	8.6 ± 3.0	5.0	0
*Trichuris trichiura*	10.0 ± 2.8	7.0 ± 1.4	12.1 ± 1.8	7.0 ± 4.2

## Discussion

The results noted in this study were compared with those reported by Tchuem-Tchuenté *et al.* [14] among other schoolchildren of Loum in 2003. According to these authors, the prevalence was 62.8% for S. haematobium, 65.5% for A. lumbricoides and 47.7% for T. trichiura, while it was 34.2%, 8.6% and 4.9%, respectively, in this study (Table 1). Similarly, egg abundance in 2003 was 54 eggs/10 mL of urine for S. haematobium, 3636 epg for A. lumbricoides and 619 epg for T. trichiura [14], whereas it was 39.6 eggs/10 mL of urine, 24.2 epg, and 10.0 epg, respectively (Table 3). These data demonstrate that the prevalence and intensity of these three helminth infections have significantly decreased over the past fourteen years and this is probably a consequence of annual deworming campaigns performed at Loum during this period to control schistosomiasis and STH infections. Preventive chemotherapy, recommended by Gabrielli *et al.* [22], is therefore effective in controlling STH infections and schistosomiasis in areas of high transmission such as Loum. Our results agree with the observations of Tchuem-Tchuenté *et al.* [11] who noted a current decline in schistosomiasis and STH infections in the Littoral region, whatever schistosome and STH species. However, another explanation based on the greater awareness of teachers and parents/guardians from these two schools to problems caused by to S. haematobium and STH infections in children cannot be completely excluded. Because of the high impact of deworming campaigns on the prevalence and intensity of these infections, our results might justify reducing the frequency of deworming campaigns, as currently recommended by World Health Organization [23].

Two other helminths were also found in the stools of several children in 2016. The prevalence of S. stercoralis (1.2%) noted in this study was within the range of values reported by Moyou-Somo *et al.* (1.62% out of 1482 pupils [24]) and Nkenfou *et al.* (0.25% out of 396 [25]) in Cameroon. In contrast, little information is available in the literature on the prevalence of T. saginata in children's stools. The authors who have worked on this parasite often mention Taenia spp. without indicating the species [26]. The prevalence of T. saginata (1.6%) noted in this study was close to that reported by Asaava *et al.* [27] in Kenyan children (2.5% out of 225 pupils) and indicated that this species should be sought, like other gastrointestinal parasites, in the stools of children, although Taenia solium was often more common than T. saginata in the latter.

According to Tchuem-Tchuenté and N'Goran [9], there was a large proportion of children carrying at least two parasite species in the schools of Loum. At the present time, polyparsitism only involved 16 pupils with 15 double infections and a single triple infection (among the 125 infected children) and has also declined sharply over the past 14 years. The three most frequent assemblages of helminths (9/16 children) in this study concerned A. lumbricoides, S. haematobium and T. trichiura and this finding is consistent with the report of Tchuem-Tchuenté *et al.* [11]. The presence of three children co-infected with S. haematobium and S. mansoni suggested that their co-infection probably occurred in the same watering place. If this explanation is valid, it would indicate that the snail hosts of both schistosome species also live there.

## Conclusion

STH infections and schistosomiasis in 2016 showed a sharp decrease in their prevalence and intensity compared to the values reported in the children of Loum by Tchuem-Tchuenté *et al.* [14] in 2003. This result confirms the trend that Tchuem-Tchuenté *et al.* [11] described in the various regions of Cameroon in 2013. The confirmation of this fall in the characteristics of these human infections would allow considering spacing in deworming campaigns among these schoolchildren, as currently recommended by the World Health Organization [23].

### What is known about this topic

- Since 2003, the city of Loum in Cameroon is known to be a highly endemic area for human *Schistosomiasis* and STH infections;
- Regular examinations of urine and stool samples, followed by annual or biannual deworming campaigns, were carried out to monitor the development of these infections among schoolchildren.

### What this study adds

- In 2016, both types of *Schistosomiasis* and STH infections among schoolchildren showed a marked drop in prevalence and intensity;
- This net decrease in the characteristics of these infections allows to propose a spacing in the monitoring of schoolchildren and campaigns of deworming.

## Competing interests

The authors declare no competing interests.
